# Supplementary material for: Translation, Adaptation, and Validity of the Short Food Literacy Questionnaire for Brazil
Source: Foods. 2022 Dec 8;11(24):3968. doi: 10.3390/foods11243968 (PMC9777863; doi:10.3390/foods11243968)
Supplement: Supplementary file 1 [file foods-11-03968-s001.zip › foods-1954323-supplementary.pdf]

Table S1: Person's correlation among the SFLQ-Br itens

Itens	Itens	Pearson's r	p
1	2	0.206	0.030
1	3	0.229	0.016
1	4	0.267	0.005
1	5	0.271	0.004
1	6	0.222	0.019
1	7	0.200	0.04
1	8	0.275	0.003
1	9	0.267	0.005
1	10	0.290	0.002
1	11	0.124	0.195
1	12	0.278	0.003
2	3	0.370	<0.001
2	4	0.334	<0.001
2	5	0.251	0.008
2	6	0.390	<0.001
2	7	0.139	0.145
2	8	0.338	<0.001
2	9	0.393	<0.001
2	10	0.303	0.001
2	11	0.354	<0.001
2	12	0.283	0.003
3	4	0.719	<0.001
3	5	0.687	<0.001
3	6	0.477	<0.001
3	7	0.447	<0.001
3	8	0.422	<0.001
3	9	0.462	<0.001
3	10	0.461	<0.001
3	11	0.446	<0.001
3	12	0.406	<0.001
4	5	0.823	<0.001
4	6	0.390	<0.001
4	7	0.385	<0.001
4	8	0.434	<0.001
4	9	0.456	<0.001
4	10	0.444	<0.001
4	11	0.427	<0.001
4	12	0.387	<0.001
5	6	0.377	<0.001
5	7	0.390	<0.001
5	8	0.315	<0.001
5	9	0.440	<0.001
5	10	0.394	<0.001
5	11	0.420	<0.001
5	12	0.350	<0.001
6	7	0.221	0.020
6	8	0.481	<0.001
6	9	0.523	<0.001
6	10	0.483	<0.001

6	11	0.483	<0.001
6	12	0.458	<0.001
7	8	0.272	0.004
7	9	0.371	<0.001
7	10	0.338	<0.001
7	11	0.318	<0.001
7	12	0.400	<0.001
8	9	0.555	<0.001
8	10	0.532	<0.001
8	11	0.352	<0.001
8	12	0.381	<0.001
9	10	0.742	<0.001
9	11	0.454	<0.001
9	12	0.444	<0.001
10	11	0.522	<0.001
10	12	0.526	<0.001
11	12	0.612	<0.001
